# Federation of Internet of Things Testbeds for the Realization of a Semantically-Enabled Multi-Domain Data Marketplace

**DOI:** 10.3390/s18103375

**Published:** 2018-10-10

**Authors:** Luis Sánchez, Jorge Lanza, Juan Ramón Santana, Rachit Agarwal, Pierre Guillaume Raverdy, Tarek Elsaleh, Yasmin Fathy, SeungMyeong Jeong, Aris Dadoukis, Thanasis Korakis, Stratos Keranidis, Philip O’Brien, Jerry Horgan, Antonio Sacchetti, Giuseppe Mastandrea, Alexandros Fragkiadakis, Pavlos Charalampidis, Nicolas Seydoux, Christelle Ecrepont, Mengxuan Zhao

**Affiliations:** 1 Network Planning and Mobile Communications Lab, Universidad de Cantabria, Edificio de Ingeniería de Telecomunicaciones, Plaza de la Ciencia s/n, 39005 Santander, Spain; jlanza@tlmat.unican.es (J.L.); jrsantana@tlmat.unican.es (J.R.S.); 2 MiMove Team, Inria Paris. 2 Rue Simon Iff, 75012 Paris, France; rachit.agarwal@inria.fr (R.A.); pierre-guillaume.raverdy@inria.fr (P.G.R.); 3 Institute for Communication Systems, University of Surrey, James Clerk Maxwell Building, Guildford, Surrey, Guildford GU2 7XH, UK; t.elsaleh@surrey.ac.uk (T.E.); y.fathy@surrey.ac.uk (Y.F.); 4 Korea Electronics Technology Institute (KETI), Seongnam 13509, Korea; sm.jeong@keti.re.kr; 5 Network Implementation Testbed Lab, Univesity of Thessaly, Volos 38221, Greece; ardadouk@gmail.com (A.D.); nasoskor@gmail.com (T.K.); 6 Gridnet, Volos 38222, Greece; sk@gridnet.gr; 7 Telecommunications Software & Systems Group, Waterford Institute of Technology, Waterford X91 P20H, Ireland; pobrien@tssg.org (P.O.); jhorgan@tssg.org (J.H.); 8 Tera s.r.l., Conversano 70014, Italy; antonio.sacchetti@terasrl.it (A.S.); giuseppe.mastandrea@terasrl.it (G.M.); 9 Institute of Computer Science, FORTH, Heraklion GR-700 13, Greece; alfrag@ics.forth.gr (A.F.); pcharala@ics.forth.gr (P.C.); 10 Laboratory for Analysis and Architecture of Systems, CNRS, 7, avenue du Colonel Roche BP 54200 31031 Toulouse CEDEX 4, France; nseydoux@laas.fr (N.S.); christelle.ecrepont@laas.fr (C.E.); 11 Easy Global Market, Le Thalassa, Bat B, 444 Route des Dolines, Valbone 06560, France; mengxuan.zhao@eglobalmark.com

**Keywords:** data marketplace, federation, Internet of Things, testbeds, platform

## Abstract

The Internet of Things (IoT) concept has attracted a lot of attention from the research and innovation community for a number of years already. One of the key drivers for this hype towards the IoT is its applicability to a plethora of different application domains. However, infrastructures enabling experimental assessment of IoT solutions are scarce. Being able to test and assess the behavior and the performance of any piece of technology (i.e., protocol, algorithm, application, service, etc.) under real-world circumstances is of utmost importance to increase the acceptance and reduce the time to market of these innovative developments. This paper describes the federation of eleven IoT deployments from heterogeneous application domains (e.g., smart cities, maritime, smart building, crowd-sensing, smart grid, etc.) with over 10,000 IoT devices overall which produce hundreds of thousands of observations per day. The paper summarizes the resources that are made available through a cloud-based platform. The main contributions from this paper are twofold. In the one hand, the insightful summary of the federated data resources are relevant to the experimenters that might be seeking for an experimental infrastructure to assess their innovations. On the other hand, the identification of the challenges met during the testbed integration process, as well as the mitigation strategies that have been implemented to face them, are of interest for testbed providers that can be considering to join the federation.

## 1. Introduction

It goes without saying that experimentation is one of the basis for technological advances [1]. Being able to test and assess the behavior and the performance of any piece of technology (i.e., protocol, algorithm, application, service, etc.) under real-world circumstances is of utmost importance to increase the acceptance and reduce the time to market of these innovative developments. However, despite the attention that the Internet of Things (IoT) has attracted [2] thanks to the large number of application domains where it can play a game changer role [3,4,5,6,7], there is still a lack of real, large-scale testbeds where all these innovations can be actually assessed. In this respect, real-life experimentation should play a major role in these developments. It would be necessary to deploy, maintain and open to the research community this kind of infrastructures so that their research can have tangible results from real-world deployments [8,9].

Interestingly, there are initiatives that, in order to improve these solutions’ maturation and significant rollout, try to support the evaluation of IoT solutions under realistic conditions in real world experimental deployments [10,11,12]. However, still they tend to lack the necessary scale or they fail to fulfil some key indicators [13,14]. Nonetheless, large-scale infrastructures enabling the assessment of developed solutions under real-world circumstances are scarce and are not always available for those willing to test their innovations. Moreover, such infrastructures are typically bound to a specific application domain, thus, not facilitating the testing of solutions with a horizontal approach (i.e., fulfilling requirements from different application domains). Finally, even when, through the combination of different testbeds, the requirements from an experimenter were fulfilled, it is necessary to deal with interoperability issues resulting from the different interfaces and models used by the different testbeds.

This paper describes the federation of testbeds that have been created within the framework of the H2020 FIESTA-IoT project [15]. The platform designed in the project provides the tools and techniques for building applications that horizontally integrate silo platforms and applications. The semantic interoperability of the diverse sensor clusters and IoT networks federated is based on the virtualization of sensors in the cloud. At the heart of these virtualization mechanisms is the modelling of heterogeneous IoT devices according to a common ontology. However, the detailed description of the platform design principles and building blocks is out of the scope of this paper. Specific insights about the EaaS IoT Platform designed and implemented in the FIESTA-IoT Project can be found at [16].

The main aim of this paper is to present the actual federation of testbeds on top of a running instance of this platform. This federation aggregates and ensures the interoperability of data streams stemming from eleven different IoT deployments. The paper summarizes the resources that are made available from eleven IoT deployments from heterogeneous application domains (smart cities, maritime environment monitoring, smart building, crowd-sensing, smart grid, smart agriculture) with over 10,000 IoT devices overall which produce hundreds of thousands of observations per day. Detailed information is provided on the amount of sensors and observations that are made available to the interested experimenters.

The main contributions from this paper are twofold. On the one hand, the paper provides an insightful summary of the federated data resources. While some informational details about the papers is publicly available (The FIESTA-IoT Testbeds. http://fiesta-iot.eu/index.php/fiesta-testbeds/ [Online 13 August 2018]), the review presented in this paper provide the necessary insights to fully understand the experimentation possibilities raised by having access to all the federated resources. This detailed and precise overview is particularly relevant to the experimenters that might be seeking for an experimental infrastructure to assess their innovations. In this sense, to the best of our knowledge, there is no single or federated IoT experimentation infrastructure currently offering the amount of data resources described in this paper. On the other hand, the paper discusses the most relevant challenges that have been met during the testbed integration process, as well as the mitigation strategies that have been implemented to face them. This discussion summarizes the technical solutions adopted to provide semantic and functional interoperability among the heterogeneous underlying IoT infrastructures and should be of particular interest for testbed providers that can be considering to join the federation. Thus, the paper goes beyond the plain description of data offerings but also presents practical insights on how the main technical challenges involved in the semantically-enabled federation of IoT platforms have been tackled. In this sense, it is important to highlight that the technical discussion presented in the paper recaps on lessons learnt from actual deployment and development experience.

The remaining of the paper is organized as follows: Section 2 briefly reviews existing related work. A high-level overview of how the testbeds have been federated and the requirements imposed by the underlying FIESTA-IoT Platform for allowing new testbeds to federate are introduced in Section 3. Section 4 presents the summary of the testbeds’ federation as well as the key details of each of the testbeds within the federation. Lessons learnt during the federation process and some discussions about the testbeds’ federation concept are sketched in Section 5. Finally, Section 6 concludes the paper.

## 2. Related Work

This section reviews on the key aspects and technologies that are behind the developments described in the paper to position the research and contributions of the paper in context and to validate its relevance by establishing the similarities and highlighting its differentiating features with currently existing approaches.

### 2.1. Experimental Infrastructures

The required effort and cost for creating realistic environments over which new IoT solutions and technologies could be tested has led to the creation of experimental testbeds open to the research and innovation community. Wisebed [17], FIT IoT-Lab [11], Fed4FIRE [18], BonFIRE [19] and GENI [20] are all testbeds that support wireless sensor network experimentation. They allow testing of new communication protocols and wireless communications that underpin the IoT domain. However, these experimental infrastructures are technology specific and mainly focused on Wireless Sensor Networks rather than on IoT as a whole. As a consequence, they do not support experimentation of new IoT applications and services. In response, SmartSantander [10] provides a large-scale, geographically distributed range of real-world sensors to test new innovative IoT services; LiveLab [21] offers a facility to evaluate human-usage of the technologies; and [22] presents a Mobile Sensing testbed of smart phones to support field-testing of new crowd-sourcing applications. Even if the usefulness of these experimental infrastructures in their own right is out of any doubt, these higher-layer testbeds are all domain specific. Thus, they do not consider an important aspect that is currently at sake within the research community. Achieving interoperability across domain silos and heterogeneous technologies is the next frontier that has to be overcome to unleash the IoT foreseen potential. The FIESTA-IoT facility is technology and domain agnostic (federating multiple smart city, smart home, crowd-sensing testbeds) to allow experiments that demonstrate IoT interoperability across highly heterogeneous IoT environments.

### 2.2. Experimentation-as-a-Service

Experimentation-as-a-Service (EaaS) model can be seen as an instance of the general Everything-as-a-Service paradigm [23] introduced and developed by leveraging the advances in cloud computing. EaaS enables stakeholders belonging to the research community or industrial sector to promote and accelerate innovation by testing and verifying new technologies supported by realistic and specialized experimental testbeds, through the use of a cloud environment. In [24] a platform for assessing the performance and reliability of commercial mobile broadband networks is presented, where authenticated users are able to orchestrate network experiments following an EaaS paradigm. FLAME project [25] is developing an EaaS architecture for exploring the viability of Future Media Internet platforms in smart-city deployments. The experiments aim to highlight the benefits of a software-enabled communication infrastructure for the optimal information distribution and control. While the FLAME project is not related with IoT, it develops an EaaS architecture for content distribution aimed at the support of experimentation and innovation on top of open platforms. This platform is similar to the FIESTA-IoT platform on top of the testbed federation presented in the paper does.

The EaaS model applied to the IoT domain stands out as an upgrade to the Sensing-as-a-Service [26] model; EaaS services are not confined to a number of virtualized sensor queries but implement and execute complex experimental workflows “in the wild” by orchestrating various devices and diverse testbeds. Thus, it is possible to widen the scope and complexity of the IoT applications supported as well as reuse the infrastructures and data streams provided and maximize their utilization. The SmartCampus facility [27] offers a testbed based on a three-tier architecture for executing user-centric experiments in an office environment, set up by external stakeholders. Although adopting the EaaS paradigm, the facility is highly domain specific in stark contrast to the FIESTA-IoT platform. A federation of experimentation platforms is presented in [28], where users are able to rapidly deploy reproducible experiments related to several smart city domains, by on-demand access to virtual reusable resources. Organicity [29] provides a facility for federating different smart city platforms and assessing the crowd-sensing problem by encouraging different stakeholders to identify urban challenges and tackle them by co-creating suitable experiments. These platforms bear a number of similarities with the one presented in this work. They offer virtualized access to IoT data produced by real-world IoT infrastructure. Datasets available spam over a number of different application domains. They allow experimentation through web-based APIs. Some of them have aggregated data from IoT infrastructures which are managed by different providers. Nevertheless, they neither enjoy the diversity of testbeds described here nor employ semantic web technologies for facilitating the semantic interoperability between deployed IoT infrastructures.

### 2.3. Semantic Interoperability

Semantic web technologies to query and manage information within federated cyber-infrastructures [30,31,32] are being used as a promising approach to guarantee the necessary consistency among IoT infrastructures. However, they usually only defines the framework and assesses the meta-directory service using their own ontologies [33,34]. Thus, they fail in taking into account the needs from IoT infrastructures that are already deployed. Moreover, the definition of the procedures to extend their deployments by integrating already existing ones is also missing. Additionally, some of them are still only proposed as theoretical solutions [35] which have not been implemented nor assessed. Finally, those that present some kind of assessment of their solutions’ implementation, while supporting the potential of the solution, exhibit a lack of exposure to real-life situations and actual heterogeneous testbeds, including large-scale IoT experimental infrastructures, which would show the true scalability and flexibility of the solutions. Only recently, the semantic interoperability have been explored in standardization fora [36] defining some base ontology and data catalogues that can be adopted by a large community, both already existing and forthcoming. The approach followed, mainly by oneM2M base ontology, is similar to the one followed within the FIESTA-IoT project for defining the FIESTA-IoT ontology [37]. The main reason for defining our own ontology was that at the time of initiating our work, the oneM2M standard was not available. However, the same that it has been possible to federate one oneM2M-based testbed, it is possible to define and implement the adaptations in the opposite direction.

### 2.4. IoT Data Marketplaces

The data generated by IoT devices is mostly owned by device owners and is often private in nature. There are however third parties that could benefit from using that data, and the challenge is in allowing them to access it under the conditions that data owners find acceptable. The concept of data marketplace has been introduced already some years ago [38]. However, although data management was clearly identified as one of the key challenges for IoT [39], solutions proposed until recently focused on creating the backend storage solutions plus the indexing mechanisms to discover the relevant pieces of information among the massive amount of data that IoT infrastructures can generate [40,41,42]. Nevertheless, there is an important requirement that has to be fulfilled. Developers who want to use existing platforms need to negotiate access individually and adapt to the platform-specific API and information models. Having to perform these actions for each platform often limits the applicability of the developed applications as they have to be tailored to the different platforms. This fragmentation of the IoT and the missing interoperability result in high entry barriers for developers and prevent the emergence of broadly accepted IoT ecosystems. Only recently some initiatives have appeared defining not only the data management platforms that support the marketplace but also the interoperability mechanisms that make the proposed solutions prepared to integrate a potentially endless amount of IoT heterogeneous infrastructures [36,43,44,45]. Although these approaches have multiple commonalities with the federation of IoT testbeds that is described in this paper, to the best of our knowledge, none of them shows the diversity and scale of the one presented in this paper.

### 2.5. Sensor Web Enablement and Web of Things

The Sensor Web Enablement (SWE) initiative (http://www.ogcnetwork.net/swe) has developed a suite of standards that can be used as building blocks for a Sensor Web. SWE defines the term Sensor Web as “Web accessible sensor networks and archived sensor data that can be discovered and accessed using standard protocols and application programming interfaces” [46]. To achieve this, SWE incorporates models for describing sensor resources and sensor observations. Further, it defines web service interfaces leveraging the models and encodings to allow accessing sensor data, tasking of sensors, and alerting based on gathered sensor observations. Leveraging the SWE concepts, the Web of Things (WoT) emerges from applying web technologies to the IoT to access information and services of physical objects. In WoT, each physical object possesses a digital counterpart. These objects are built according to Representational state transfer (REST) architecture and accessed with HTTP protocol via RESTful API. A Web Thing can have an HTML or JSON representation, REST API to access its properties and actions, and an OWL-based semantic description.

W3C has recently launched the Web of Things Working Group [47] to develop initial standards for the Web of Things. Its main aim is “to counter the fragmentation of the IoT”. They are still working on defining the WoT architecture and the description of the WoT Thing, which should define a model and representation for describing the metadata and interfaces of Things, where a Thing is the virtualization of a physical entity that provides interactions to and participates in the WoT.

In parallel to this standardization efforts, several projects and platforms have been developed targeting the support of service provision based on the SWE and WoT paradigms. In [48], authors present their WoT-based platform for the development and provision of smart city services. Precision agriculture is the application domain which benefits from the platform described in [34]. While they provide some of the solutions promised by the WoT, still do not address the IoT fragmentation as they rely on proprietary modelling. In the works presented in [49,50,51], semantic technologies are employed to fulfil the extendable modelling requirement. As we are proposing in this paper, we believe that this is the necessary combination in order to fully develop the WoT concept into a running system. The key novelty from the work presented in this paper is that, most of the existing previous works have not been implemented and proven over real-world scenarios with federation of heterogeneous IoT infrastructures, as it is the case of the platform presented in this paper. Some well-known implemented Sensor Webs [52,53,54], which have actually put SWE and WoT standards and paradigms in practice, have not faced the necessary heterogeneity to actually face the interoperability challenge.

### 2.6. Semantic Web and Semantic Annotation of Sensor Data

In semantic web, there exists standards such as SSN ontology [55] that are widely accepted and used by different testbeds. A new version of SSN ontology [56] covers mainly concepts that are common to most of the domains (horizontal concepts). SSN ontology defines concepts such as Sensor, Observation, Samples and Actuators that form core concepts within the Domain and Information Models defined in the IoT Architecture Reference model (ARM) [57]. However, in more abstract terms, definitions of such concepts are provided in another widely promoted oneM2M ontology [58]. There are several other ontologies such as Smart Appliance REFerence (SAREF) [59] that are standardized but are context-based and only focus on one domain (vertical concepts). Efforts have been made to unify concepts defined vertically and horizontally so that federation and interoperability can be achieved. One such effort is that of lightweight FIESTA-IoT ontology [37] that reuses concepts to focuses on competency based questions [60] across different domains. It mainly targets concepts that are necessary to answer: who is the provider of the information, under what conditions observations are collected, when and where are the observations made and how collected information is exposed. In FIESTA-IoT ontology the core concepts are borrowed from SSN and IoT-lite ontology [61], a lighter version of the IoT-A ontology [62], to conform with IoT ARM’s Domain and Information Models. These core concepts relate to defining:

- A Resource which is a “Computational element that gives access to information about or actuation capabilities on a Physical Entity” [57].
- An IoT service which is a “Software component enabling interaction with IoT resources through a well-defined interface.” [57].
- An Observation is an “Act of carrying out a procedure to estimate or calculate a value of a property of a feature of interest” [63].

Further many related concepts to abovementioned core concepts have been reused in FIESTA-IoT ontology. As it has been already said, the FIESTA-IoT ontology is defined to be as lightweight as possible precisely to cope with this requirement of easing the integration process. To keep it lightweight, on top of the core concepts FIESTA-IoT ontology reuses some of the already defined concepts and relationships between the concepts. To name a few, these are: Platform defined via SSN, Instant defined via Time [64], QuantityKind and Unit defined through the M3-Lite taxonomy [65], Point defined through WGS84 [66], Metadata defined using IoT-lite, hasDataValue defined using DUL [67], hasDomainOfInterest defined using M3-lite taxonomy and location defined using WGS84.

## 3. Testbed Federation Concept and Conditions

The main aim of the FIESTA-IoT federation is to enable an EaaS paradigm for IoT experiments. However, instead of deploying yet another physical IoT infrastructure it enables experimenters to use a single EaaS API for executing experiments over multiple existing IoT testbeds that are federated in a testbed agnostic way [16]. Testbed agnostic implies in this case the ability to expose a single testbed that virtualizes the access to the underlying physical IoT testbeds. Experimenters learn once and accordingly use the EaaS API to access data from any of the underlying testbeds.

To this end, the testbeds that aim to participate in the federation have to implement the common standardized semantics and interfaces that have been defined within the FIESTA-IoT project. This enables the FIESTA-IoT meta-platform to access the data that their devices produce as well as the descriptions of their devices and the services that these devices might expose.

**Figure 1:**
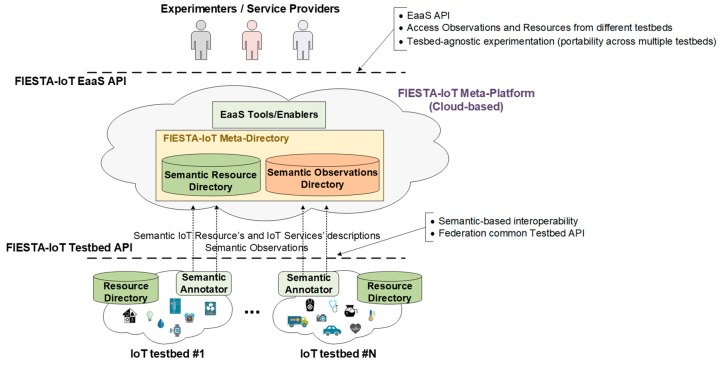
FIESTA-IoT Platform abstract EaaS and testbed federation concepts overview.

Figure 1 presents the abstract EaaS and testbed federation concepts overview for the of the FIESTA-IoT meta-platform. Its central component is a directory service (so-called FIESTA-IoT meta-directory), where sensors (or IoT resources) from multiple testbeds are registered along with the observations produced by them. This directory enables the dynamic discovery and use of IoT resources (e.g., sensors, actuators, services, etc.) from all the interconnected testbeds.

The key concept behind the federation of IoT testbeds is the specification of a common Testbed API that defines the interfaces to carry out the registration of the testbed resources as well as pushing of the observations to the meta-directory. Besides the actual technologies used for implementing these interfaces, the main feature that underlies the FIESTA-IoT Testbed API is the fact that the information is exchanged in a semantically annotated format.

In this sense, the main design decision is the use of semantic technologies to support the interoperability between heterogeneous IoT platforms and testbeds. The FIESTA-IoT ontology [37,68] makes it possible to seamlessly deal with data from different sources. The phenomena and units of measurement related concepts have been incorporated to the FIESTA-IoT ontology through the M3-Lite taxonomy. This taxonomy has been created by integrating and aligning already existing ontologies in order to homogenize the existing scattered environment in which a quite large number of similar ontologies define the same concepts in an overlapping manner.

Federated testbeds have to implement their own Semantic Annotators to transform the data they handle internally to the common semantic ontology defined by FIESTA-IoT. Different RDF representation formats (e.g., RDF/XML, JSON-LD, Turtle, etc.) are supported as long as the common ontology is used.

The FIESTA-IoT Platform takes as reference the IoT ARM as defined in the IoT-A project [57]. ARM consists of Domain and Information model that also form a base for data that is being stored in. Although, the model defines Resources [57] and IoT-Services [57], it misses to define the observations [63] collected by the IoT-devices. FIESTA-IoT ontology considers all such aspects (see Section 2) and thus enables the FIESTA-IoT architecture to be based on a canonical set of concepts that all IoT platforms can easily adopt.

The adoption of these essential concepts only requires, from the underlying testbeds, a straightforward tuning of the models that they handle internally. In this sense, independently of which internal model the testbeds uses, whether it is proprietary or based on existing standards [69,70], the Testbed Provider (TP) should be able to find in a quite straightforward manner how to map the internal modelling to the canonical concepts managed within the FIESTA-IoT ontology. The aforementioned tuning of models basically consist on mapping the internal structure of information to the one that uses the FIESTA-IoT ontology as a basis. The less number of concepts to map and the more fundamental these concepts are, the less the chances to have a TP that is unable to perform the mapping between her internal data model and the interoperable model used within the FIESTA-IoT Platform that is based in the FIESTA-IoT ontology.

The complete description of the FIESTA-IoT ontology is out of the scope of this paper. A complete specification of the FIESTA-IoT ontology is defined in [37]. It is important to emphasize that this ontology is the baseline for the interoperability of the heterogeneous testbeds and IoT platforms that have been already federated and those that will be joining the FIESTA-IoT meta-platform in the future. The different testbeds have to converge for participating in the federation and they use this ontology as the reference for this convergence. Precisely this is the main reason why the ontology has been kept simple as a design decision.

Since a testbed may internally use various standard and/or proprietary interfaces, in addition to the semantic model that underpins the interaction between each of the testbeds and the FIESTA-IoT Platform, a list of services (so-called Testbed Provider Interface—TPI) has been specified. A testbed has to expose, at least, a subset of them in order to enable different connection methods to the platform.

The TPI is a set of RESTful web services that spans across two different realms [16]. The first is the FIESTA-IoT Platform side with the TPI Configuration & Management layer that controls the functionality of the TPI. The second is the testbed side with the Testbed Provider Services (TPS) where the TP has to implement a set of services that enables the management and manipulation of the offered data. These services can be grouped into two types according to the relation established between the testbed and the FIESTA-IoT Platform, namely get-based and push-based. For the Get case, the services should respond with the latest observations from a list of sensors. For the Push case, the services correspondingly initiate a stream at the testbed side that pushes the observations from a list of sensors. In both cases, the list of sensors from whom the observations are retrieved or have to be pushed is the input parameter. The TPs can choose which of these two options fit better in their platforms and decide to either let the platform control the schedule (get-like option) or control the schedule of when to push the data (push-like option).

## 4. Federated Testbeds

This section summarizes the main characteristics and datasets available through the FIESTA-IoT Platform (FIESTA-IoT Platform. https://platform.fiesta-iot.eu. [Online 3 September 2018]) after the integration, until the time of writing this paper, of eleven different IoT infrastructures.

### 4.1. Criteria for Testbed Federation

The federation initially started with five testbeds being integrated with the platform to serve as reference implementations for testbed integration [71]. These were SmartSantander, SmartICS, SoundCity, Grasse Smart Territory and CABIN. In order to enlarge the value of the offer and also to proof the adequacy of the solutions designed to enable interoperability among heterogeneous IoT platforms, two open calls for testbed integration were conducted. As a result of these Calls, six more testbeds were selected.

The main aim of federating more IoT testbeds and not restricting it to the original four ones is to challenge the platform design. This way tuning of that design can be made by following the lessons learnt and best practices that can only be elicited from actual implementation. Moreover, addition of more application domains also brings further challenges that were not initially considered as they were not present in the initial set of testbeds. This selection was based on the following criteria:

- *Usefulness*: the degree of expected future use of the extension, which takes into account the amplitude (number and variety) of the testbed IoT resources, their nature (i.e., real or virtual resources), the testbed availability and the accessibility to the testbed resources for platform users during the whole project duration and beyond.
- *Complementarity*: the degree at which the testbed will provide new datasets and data streams, whereby it contributes to enlarge the critical mass of the existing experimentation support capacity offered by the 4 integrated testbeds, as well as to probe the interoperability solutions developed within the project, by providing additional datasets and data-streams on the domains of interest of the existing ones. Else, it can offer extra scenarios (smart agriculture, smart factory, crowd-sensing, underwater, etc.) with a high potential impact in terms of the real-world innovation enabled through the offered infrastructure and its associated datasets and data-streams.
- *Sustainability*: The guarantee of availability of the services offered by the extension in absence of future funding. This is linked with the history of the infrastructure and its demonstrable ability to support experimentation.
- *Technical competence*: The testbed provider should exhibit prior testbed management experience and the necessary qualifications to integrate their testbeds within the FIESTA-IoT federation.
- *Feedback*: The potential for providing feedback regarding the platform and the process of integrating new testbeds within the federation. Testbed providers must demonstrate value of the FIESTA-IoT federation procedures and/or motivate added-value extensions. Also, the business impact for joining the federation was considered.

**Figure 2:**
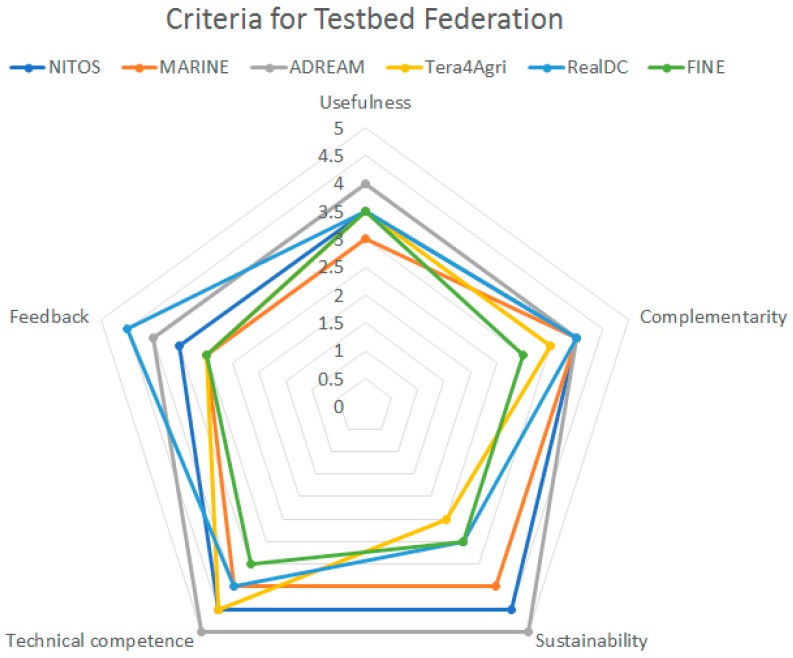
Assessment of the six testbeds that joined the federation through Open Calls process.

Figure 2 illustrates the assessment of the testbeds based on the criteria set out in the Calls processes. As it has been previously mentioned, the six testbeds whose key features are assessed in Figure 2 are those that were selected during the Open Calls process. The remaining five testbeds were the founding members of the federation.

The overall marks of the different testbeds are remarkable and interestingly high in Complementarity. This provides good insight on the heterogeneity that, in general, is exhibited by the compound offering of the federation of testbeds. This conclusion is also supported by the above average marks that all the testbeds received in the Feedback criterion. On the one hand it shows that the integration of these testbeds can generate valuable lessons learnt, which could not be elicited otherwise, and, on the other hand, it indicates, together with the respective Usefulness marks, the added-value of integrating the offerings from these testbeds.

Another criterion with several excellent marks is the Sustainability. While this is not related to the technical challenges brought forward by the inclusion of this testbed, it certainly demonstrates that the federated testbeds have a long track of previous and future IoT experimentation support.

### 4.2. Overall Federation Summary and Data Marketplace Offering

For almost all the cases, the IoT devices are actually located around the TP premises. However, since the SoundCity testbed is based on data stemming from smartphones, its actual coverage is not limited to Paris.

Table 1 provides a summary of the eleven testbeds and highlights roughly the application domain and the IoT devices that are part of each of the deployments. Further, the Table 2 presents categorization of the eleven testbed in different application domain.

Most of these testbeds are internally using proprietary platforms which does not follow any specific standard or largely adopted IoT platform. CABIN testbed is based on oneM2M standard. However, it only implements the functional specifications and not the semantic ontology that has been recently defined by the ETSI oneM2M standard. ADREAM testbed uses a proprietary ontology [76] which is certainly aligned with the oneM2M base ontology but still is not part of any standard. Finally, SmartSantander testbed is based on FIWARE (https://www.fiware.org/ [Online 3 September 2018]) generic enablers and follows the data catalogues. All in all, the heterogeneity is, significantly, the main feature that can be derived from these testbeds.

**Figure 3:**
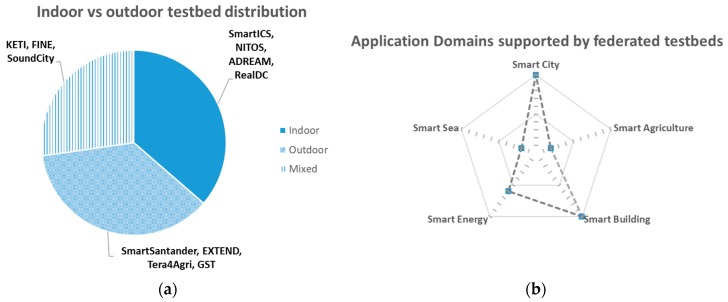
Application domains of the integrated sensors.

Overall, there is a reasonable balance between indoor and outdoor sensors (cf. Figure 3a) covering five wide application domains (cf. Figure 3b), namely smart city, smart building, smart energy, smart agriculture and smart sea). The spider graph in Figure 3b roughly represents the depth in which each of the application domains are covered attending to how many of the federated testbeds cover that particular domain. This creates a quite varied offering able to cope with the needs of a good number of potential IoT researchers and innovators which would like to assess their developments in a scenario excerpted from the combined offering.

**Figure 4:**
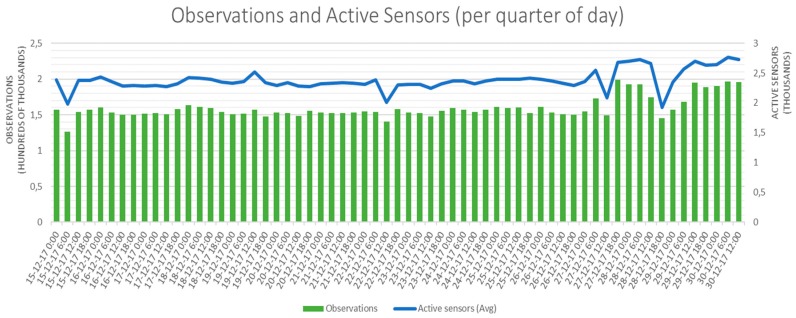
Active sensors and generated observations during December 2017.

In addition to the general details, it is important to highlight the raw figures that the federation of testbeds is continuously providing in terms of active sensors and amount of observations generated. In this sense, Figure 4 shows these two key parameters’ evolution during the second half of December 2017.

More or less constantly, every 6 hours (time scale in X-axis is set to quarter of day for the sake of clarity), the testbeds’ federation is generating around 150,000 observations. These observations are coming, on average, from 2000 active sensors that are continuously capturing events in its environment and reporting them to the platform.

**Figure 5:**
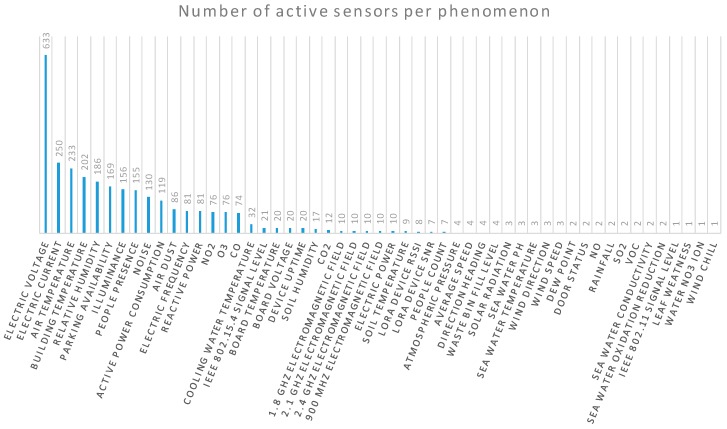
Amount of active sensors per phenomenon.

Table A1 (in Appendix A) and Figure 5 present the detailed analysis of the offerings from each of the FIESTA-IoT testbeds. In contrast with information available on-line [77], the figures summarized in Table A1 (in Appendix A) has been extracted from the actual FIESTA-IoT Platform, thus, presenting the actually available number of active sensors and their observations’ generation frequency and not the textual description of testbeds that indicates the deployed devices but not the ones regularly producing observations, which is the most relevant information for the experimenters willing to carry out their experiments on top of the FIESTA-IoT Platform. The key details presented in Appendix A, apart from the actual amount of devices producing data from each testbed, are the Phenomenon, which stands for the physical parameter observed, and the Quantity Kind, which stands for the Internationalized Resource Identifier (IRI) assigned in the FIESTA-IoT ontology to the respective physical phenomenon. This latter parameter is particularly important as it is the one that has to be used within the FIESTA-IoT Platform when looking for the corresponding phenomenon.

**Figure 6:**
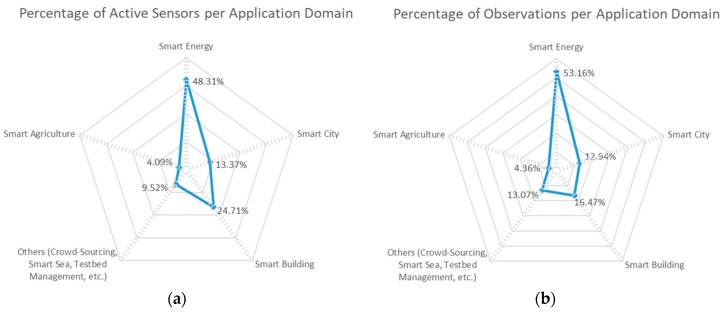
Distribution of active sensors (**a**) and observations (**b**) per application domain.

In terms of the covered application domains, as it is shown in Figure 6, the Smart Energy domain is the one that has both more active sensors and more generated observations. In this respect, the RealDC, SmartICS and ADREAM testbeds have a large set of IoT devices measuring the power consumption at its Data Centre, offices’ desks and buildings respectively, which produce observations quite often.

Following, the Smart City deployments from the SmartSantander and FINE testbeds also contribute with a significant amount of observations and active sensors to the federation offering. Testbeds, such as CABIN and NITOS, characterized under Smart Building accounts for the sensors that are deployment at indoor environments measuring environmental conditions at the different buildings at which some of the federated testbeds are deployed. Last but not least, Smart Agriculture deployments (Tera4Agri) has a smaller but still relevant share of the available offering. Finally, there are several sensors made available by various testbeds that can be catalogued under other application domains (such as CrowdSensing—SoundCity, Smart Sea—MARINE, Testbed Management or Wireless Network Status—Grasse Territory) as well and still can be discovered by a researcher and/or innovator willing to experiment with them. More yet brief description about the testbeds is provided in Section 4.3.

**Figure 7:**
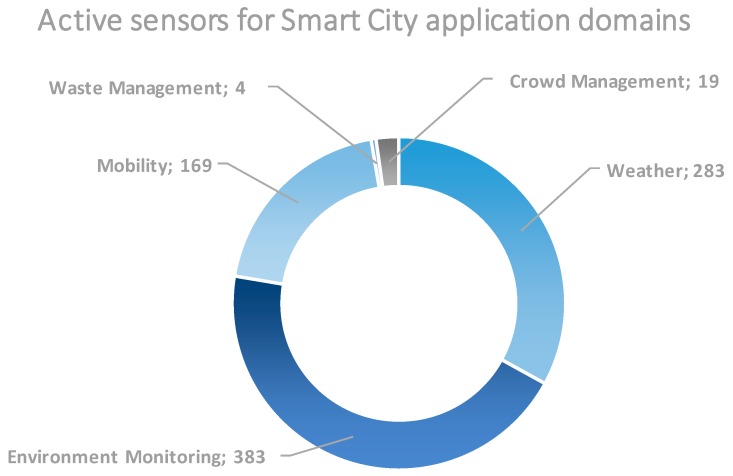
Distribution of active sensors per application sub-domain within the Smart City domain.

Interestingly, for the Smart City domain, several sub-domains (cf. Figure 7) are covered by the combined deployments. While SmartSantander deployment accounts for most of these active sensors, there are other testbeds like FINE or SoundCity that contribute with several environment monitoring sensors or CABIN that includes parking availability sensors, thus, enabling experiments that can be transparently applied to different cities.

**Figure 8:**
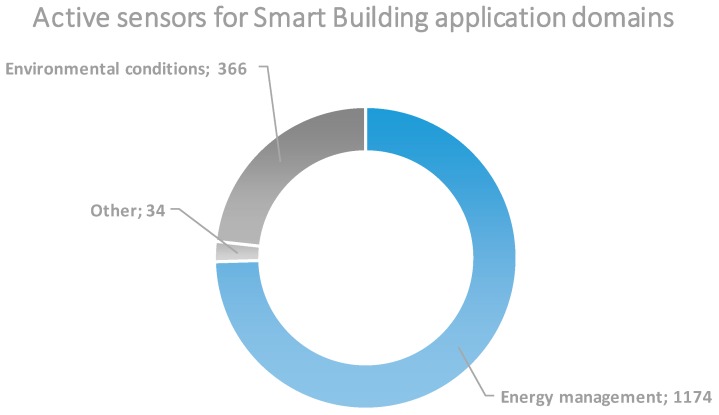
Distribution of active sensors per application sub-domain within the Smart Building domain.

The other two large domains, in terms of available active sensors, are the Smart Energy and Smart Building. They can be combined into one since they are actually building energy management sensors the ones coming mainly from RealDC and ADREAM testbeds. For this combined domain, also some internal categorization can be made as presented in Figure 8. The other testbeds related to this category, like SmartICS, NITOS and CABIN contribute to the other big sub-domain, namely, building environmental conditions.

### 4.3. Federated Testbeds Overview

One of the largest testbeds within FIESTA-IoT federation is the SmartSantander testbed. It is an experimental test facility for the research and experimentation of architectures, key enabling technologies, services and applications for the IoT in the context of a city. The infrastructure made up of 12,000 deployed diverse IoT devices covers a wide area of the city of Santander, located in the north of Spain. This testbed goes beyond the experimental validation of novel IoT technologies. It also aims at supporting the assessment of the socio-economical acceptance of new IoT solutions and the quantification of service usability and performance with end users in the loop.

SmartICS focuses on the domain of smart buildings and is deployed in the Institute of Communication Systems at the University of Surrey. It provides a facility for IoT experimentation using a variety of sensor devices deployed within the building. These sensor devices are mainly installed on office desks in the building and are used to capture a number of quantity kinds relating to air quality, ambient environment, energy consumption and desk occupancy. Observations from the devices are reported to a proprietary testbed server every minute. The proprietary testbed server keeps a register of all reporting sensor devices, and a data repository for each sensor device. Experimenters can interact with the testbed through a proprietary RESTful API, whereby sensors are exposed as dereferenceable web resources.

SoundCity testbed leverages the Ambiciti (http://ambiciti.io [Online 13th August 2018]) mobile application and the sensors within the smartphone to collect ambient noise measurements. On top of collecting such participatory data on a large scale, the Ambiciti mobile application provides its users with the ability to form groups and contribute to that specific group. The SoundCity testbed leverages such grouping feature and only federates data within the “FIESTA-IoT” group in the FIESTA-IoT platform.

CABIN (Context Aware smart BuildINg) is located on the KETI headquarter premises in Seongnam city, South Korea. This testbed is deployed using OCEAN open source software that implement a M2M IoT platform global standard. The main purpose for the deployed infrastructure is to study building energy optimization considering human behavior. In addition to the indoor sensors, parking sensors are also deployed outside the building. The benefit of having the CABIN testbed is to ensure oneM2M TPS replicability for future oneM2M and FIESTA-IoT enabled testbeds.

NITOS Future Internet Facility is an integrated facility with heterogeneous testbeds that focuses on supporting experimentation-based research in wired networks, wireless networks and IoT in general. NITOS is remotely accessible and open to the research community 24/7 and supports evaluation of protocols and applications under real world settings. The testbed is based on open-source software that allows the design and implementation of new algorithms, enabling new functionalities on the existing hardware. NITOS WSN testbed is part of the overall facility and offers several NITOS Wireless Sensor Motes developed in house by NITlab (https://nitlab.inf.uth.gr/ [Online 13th August 2018]) and deployed in an office environment. NITOS WSN is a smart building testbed, capable of measuring environmental parameters with the purpose of providing the infrastructure upon which an experimenter can build own applications.

The MARINE testbed has been deployed by GRIDNET (http://gridnet.gr/MARINE/ [Online 13th August 2018) for testing the performance of own prototype communication hardware enabling IoT applications in the marine and city environments. Testbed nodes are equipped with a wide variety of heterogeneous communication technologies, ranging from IoT related standards (ZigBee, LoRa) to the widely adopted Wi-Fi and LTE protocols, along with a unique real-time power monitoring framework for monitoring consumption of wireless interfaces. Moreover, the nodes feature a wide set of environmental sensors (17 different sensor types), suitable for application scenarios such as monitoring of water and air quality and detection of potential dangers for inhabitants of the area. The facility currently consists of 8 fixed air quality monitoring stations deployed in the city of Volos, Greece and 4 floating seawater quality monitoring buoys deployed in a bathing and recreation coastal area, close to the city.

RealDC provides live Data Centre environmental information into the FIESTA-IoT ecosystem. This integration comes in the form of power consumption, cooling and ambient weather. The data is captured at five-minute intervals.

Tera4agri Testbed comprises of data collected from the monitoring of environment, soil and trees. Thereby enabling the implementation of innovative experiments in the agriculture domain. The testbed is located in Minervino Murge (Italy) in the Tormaresca - “Bocca di Lupo” (one of Italy’s top wineries) estate. The testbed collects data from the sensors using the Tera s.r.l’s Internet of Everything Gateway.

FINE facility provides an experimental testbed that is able to support innovative IoT applications in the smart city domain. It utilizes RERUM architecture [78] for enabling the interconnectivity of a large number of heterogeneous IoT devices based on the concept of security, privacy and reliability by design. The testbed comprises several indoor and outdoor deployments in the city of Heraklion in Crete, Greece, operating on 6LoWPAN and LoRaWAN communication technologies to aid applications such as environmental monitoring, comfort quality and energy management and smart parking.

The Grasse Smart Territory Testbed is an experimental testbed for Smart City applications for the urban, suburb and rural areas of the City of Grasse. The main purpose of the testbed is to provide experimental digital facilities and applications to the citizens to make life greener and more efficient using state-of-the-art IoT technologies, and to make public authorities’ managers understand the way IoT technologies can benefit to citizens. It is developed with the collaboration of the local authorities and other local associations and companies. The privileges are given to the use of LoRa technology for the connectivity of devices, which can significantly extend the battery life on the field devices. Several environmental sensors, i.e., CO2, pollen, humidity, are being deployed and tested to be connected to the testbed.

The ADREAM building is a living lab providing a horizontal platform to foster research projects, either focused on one aspect of the building or cross-domain. The building is meant to have as little energy footprint as possible and is thus equipped with a large range of sensors to analyze its energy consumption, as well as its production based on solar panels.

## 5. Federation Process Discussion

Interoperability is at the core of many IoT applications [79]. That is what the FIESTA-IoT platform, with its unified interface and vocabulary enabling access to testbeds data, adds value to said data by improving its interoperability. However, the original value remains within the data itself, collected by each testbed individually and unified by the platform. Therefore, the value is created by the testbeds, and improved by the platform. This section of the paper aims at describing the scientific and technical challenges met during the integration of the currently federated testbeds, in order to pave the way for future testbeds and ease their integration.

The integration of a testbed within the platform is a well-defined process, constituted of a series of steps [16]:

- The testbed’s data model is aligned to the FIESTA-IoT taxonomy;
- The testbed provider develops an annotator to enrich the data, and a TPS to expose it;
- The compliance of the annotated data and of the TPS are examined, and the testbed is certified;
- The testbed and its resources (sensors) are registered on the platform;
- The testbed provider configures the data collection process.

Data arriving to the FIESTA-IoT Platform from the testbeds has to be semantically annotated using the FIESTA-IoT Ontology. So, for the first step TPs can either develop the annotator themselves or use the AaaS API provided by the FIESTA-IoT platform. After successfully generating the testbed’s annotator, the TP should decide on how the captured observations are going to be provided and develop the TPS accordingly. The two possibilities are to instruct FIESTA-IoT Platform to periodically poll the testbed for the observations generated or to directly push observations into the FIESTA-IoT Platform as they are generated. In order to facilitate the TP with the TPS development, FIESTA-IoT provides a skeleton component in Java (Java Software Development Kit: http://www.oracle.com/ technetwork/java/index.html [Online 13 August 2018]) implementing all the services required by the FIESTA-IoT Platform. From this skeleton, the TP would only need to develop the testbed’s internal data access and annotator integration. Once the TPS implementation is completed, the next step would be to validate the implemented TPS and annotator. The FIESTA-IoT Platform includes a Certification Portal that can be used by the TP to get FIESTA-IoT certified. Moreover, the tools available on this Certification Portal can help the TP in the previous two steps. The next step would be to register the available testbeds and resources to the FIESTA-IoT Platform. The TP can make use of the tools at the FIESTA-IoT Platform Portal UI for this process. Finally, the TP should instantiate and schedule the data pushing (testbed controls the scheduling) or retrieval (platform polls periodically).

In the remainder of this section, the challenges met at each step of this integration process are described, as well as the mitigation strategies that have been implemented to face them. Indeed, model alignment, data transformation or massive data publication are not trivial topics, present in many IoT deployments [80] beyond the FIESTA-IoT platform. The description of these challenges and their mitigations is followed by a discussion on the benefits for the TPs who joined the federation.

### 5.1. Technical Requirements Discussion

This section discusses the key lessons learnt and best practices that the experience of integrating the previously described testbeds within the FIESTA-IoT Platform has allowed us to extract.

#### 5.1.1. Preparing the Integration

The testbeds are completely independent of each other, and the architectures they deploy are highly heterogeneous. The purpose of the integration process described in this section is to lead these initially unrelated data providers into compliance with the FIESTA-IoT platform interface, so that the produced data is seamlessly consumable by the experimenters. At the end of the process, the testbed is certified and is integrated into the federation.

##### Data Model Alignment

The data within FIESTA-IoT platform is annotated with a single vocabulary, described using M3-lite taxonomy [65]. Initially, as there is no constraint whatsoever on the testbeds internals, the data model used by the testbeds are very diverse, and range from relational databases to datasets semantically annotated with an ad-hoc vocabulary. The first step to providing interoperability over the testbed data is to align its idiosyncratic data model to the common vocabulary.

Challenges and mitigation: One of the challenges met at this point in the federation is that some subjective modelling choices do not allow a straightforward one to one equivalence mapping between a testbed model and the target vocabulary (especially when a testbed has its own vocabulary). Some elements implicit on the testbed side had to be made explicit to be compliant with the platform, and knowledge that was stored in a several separate knowledge bases had to be gathered in a single repository. As one of the mitigation strategies, FIESTA-IoT platform provides a dedicated workflow for testbeds to propose updates to the vocabulary with the needed concepts.

Specific technical details: As it has been already described, the FIESTA-IoT ontology has been designed for the basic modelling of any IoT system. This has been proven correct since all the 11 testbeds were able to accommodate to this basic modelling. However, while the core of the ontology has remained stable, the addition of testbeds to the federation has resulted in extensions to the ontology in terms of *QuantityKind* and *Unit*. These two classes are the parent classes used for describing the physical phenomenon observed and the unit of measurement used respectively. These classes are key both for describing the sensors and actuators that conform an IoT deployment as well as the observations that they generate. In this sense, while the core ontology uses the parent classes (i.e., *QuantityKind* and *Unit*), any sub-class from them can be used for describing IoT devices and observations. The workflow used to progressively update the FIESTA-IoT ontology basically consisted in requesting the TPs to make an initial study of the FIESTA-IoT ontology and only if none of the already existing sub-classes of *QuantityKind* and *Unit* met some of their needs, this is, one of their IoT devices observes a physical phenomenon not yet covered, they could make an argued request for addition. The requests for additions coming from the TPs were subsequently analyzed by the FIESTA-IoT ontology maintainers and incorporated if they were necessary. This process was straightforward in most of the cases. Only some iterations were necessary when the arguments incorporated to the request for addition were not clear enough. Most of the times the TPs seamlessly found the appropriate *QuantityKind* and *Unit* sub-classes that their testbeds needed.

##### Building a Resource Description

The FIESTA-IoT platform hosts the description of the devices that are associated to a testbed. Once the data model is aligned it is essential that resources are described in the needed format and are registered to the FIESTA-IoT platform. The FIESTA-IoT platform provides a tool to generate said descriptions in compliance with the vocabulary with a capability to register one device at a time or perform bulk upload if the annotations are already available.

Challenges and mitigation: As said in the previous section, testbeds that use semantics internally to store data have to comply with the unified FIESTA-IoT model. The generation of such mapping could be time consuming effort. For testbeds that already have a semantic description of their devices, the approach proposed by [81] allows the translation of the source description into the target using the alignment between data models. Moreover, for testbeds that do not have semantic descriptions at hand, using AaaS tool, a testbed can generate annotation of the resource description and can use them to register the resources. The services therein have capability to provide annotated descriptions to both resources and observations based on information that is provided to it post the data model alignment phase. On top of AaaS tool, FIESTA-IoT also provides best practices that a testbed should follow while producing annotations to harmonize and ease out building annotations.

Specific technical details: The AaaS tool leverages the baseline structure of the FIESTA-IoT ontology and the design criteria followed during its definition. In this sense, for generating the annotated version of their IoT devices, the TPs could directly take the FIESTA-IoT ontology and the examples provided in the handbook (FIESTA-IoT Handbook. http://moodle.fiesta-iot.eu/pluginfile.php/711/ mod_ resource/content/6/FIESTA-IoT_Handbook4ThirdParties_v4.3.pdf) provided as training material, which provides best practices for producing valid annotations. However, it is highly recommended to make use of the AaaS tool. Not only because, it eases the process but also because it guarantees validity and certification of the testbed. In terms of IoT devices descriptions, the AaaS tool followed the cardinality included into the FIESTA-IoT ontology and defined through a JSON schema the mandatory attributes that must be included by the TPs for requesting the annotated description of one of their sensors. Basically, it requested a valid unique identifier, and its sensing and/or actuating capabilities. This latter aspect included the type of sensor and the physical phenomenon observed (or actuated upon). TPs were asked to provide the corresponding *SensingDevice*, *QuantityKind* and *Unit* sub-class from the FIESTA-IoT ontology. Additionally, the TPs had to include their testbed unique identifier in order to internally link their devices to their testbeds. Apart from the mandatory fields, the TPs could enrich the descriptions of their devices with additional optional attributes. For example, the device location, which was not mandatory to support mobile devices, or some metadata about the device sensing capacities (e.g., its accuracy, frequency, etc.). The AaaS tool compiled the information provided by the TP through a JSON document, posted to the RESTful AaaS’ API, and generated the corresponding RDF document. The AaaS allowed to generate the description of many devices at once.

##### Annotating Observation Data

Providing a unified access to resource observations requires said observations to be annotated with the common vocabulary. This annotation can either be performed by the testbed if it has semantic capabilities, or by the FIESTA-IoT platform using the AaaS tool mentioned in the previous section.

Challenges and mitigation: As stated before, within FIESTA-IoT, there are different testbeds that are associated with different application domains of IoT. Some testbeds such as SoundCity, SmartICS, and ADREAM face privacy challenges when it comes to publishing data to open platforms such as FIESTA-IoT. In order to publish data, such testbeds have to first get consent of users before the user specific data can be published. Further, testbeds have to anonymize the data so that there is enough noise when correlation of the published data sample to a particular user is performed. Privacy also pose challenge when data attributes are not reported due to privacy concerns. This make it hard for testbeds to comply with the FIESTA-IoT ontology causing annotations to fail the validation step. To mitigate this problem, testbeds should set default value for the data attributes that are not reported.

Specific technical details: Similarly to the case of annotating the description of the testbed resources, the AaaS tool offered the possibility to produce valid RDF documents for the observations generated by that IoT devices. In this case, the mandatory fields, according to the concept’s cardinality defined by the FIESTA-IoT ontology, were, apart from the observation value, the observation location, its timestamp, the identifier of the sensor that produced it and the physical phenomenon observed, together with the unit of measurement. As it happened with the device descriptions, these mandatory fields were available for all the testbeds so the integration was seamless. It is important to highlight that this is only possible because of the appropriate design of the FIESTA-IoT ontology, which has demonstrated its flexibility and scalability to accommodate the integration of the eleven testbeds.

While the use of the AaaS for the generation of annotated resource descriptions is a process that would not be so frequent (only at the initial testbed integration and when new IoT devices are deployed at the testbed), its use for generating valid RDF-formatted observations had important delay restrictions. The frequency with which a testbed produces an observation can be really big. Thus, the AaaS should not increase the processing time too much. The implementation of the AaaS kept the annotated observation generation time in the range of some milliseconds.

##### Implementing the TPS

The platform endpoint is publicly known, but each testbed has its own domain name and public interfaces. To make it possible for the platform to collect data from the multiple testbeds, each testbed has to implement on of the services from its TPS. From an architectural standpoint, the TPS is an interworking proxy, hiding the internal specificities of each testbed under a common interface. Using the TPS, the FIESTA-IoT platform is able to either actively collect data (pull-based approach) or to subscribe for push-based publication initiated by the testbed. Implementing the former or the latter is up to the testbed provider, depending on which is more convenient considering its own workflow.

Challenges and mitigation: In order to enable communication between the testbed and the platform, access credentials have to be provided and right HTTP certificates have to be set. While different identification methods are supported by the FIESTA-IoT Platform, the integrated testbeds only used two of them: API Key over HTTPS connection and IP filtering. Indeed, only the first one impose some requirements on FIESTA-IoT Platform while the second is transparently handled at the testbed side. Being able to identify the issuer of a request to the testbed is a security issue, as it prevents malicious requests to be processed.

Specific technical details: After successfully generating the testbed’s annotator the TPs had to functionally integrate the two domains at hand, this is, their testbeds and the FIESTA-IoT Platform. For this functional integration, the TPI specification considers the main functionalities and properties that should be exposed by IoT testbeds in order to enable their integration within the EaaS Platform for the purposes of testbed-agnostic experimentation. The TPI spans across two different realms. On the one side, the testbed side, the TPS API have to expose a set of services that enables the management and manipulation of the offered data. At the other side, the FIESTA-IoT Platform side, the behavior of the TPS methods is controlled from a set of services so-called TPI Data Management Services (DMS). They enable the TPs to consume and control the TPS services that their testbeds expose either by identifying a specific schedule or by enabling a data stream connection. The TPs should decide on how the captured observations are going to be provided, this means if the “Get” or the “Push” methodology is going to be used, and develop the TPS accordingly. In order to facilitate the TP with the TPS development a skeleton component implementing all the required services has been provided. This skeleton only requires the testbed’s internal data access and annotator integration.

##### Obtaining a Certification

At this point, all the functionalities required for the testbed to be integrated to the federation are implemented. To validate this implementation, the testbed must go through a certification process available on the platform where the compliance of both resources and observations description are evaluated along with the conformance of the TPS behavior with the specification.

Challenges and mitigation: Generally this step was not imposing any challenge in the process. Indeed, as TPs are able to access the tools available on the Certification Portal (FIESTA-IoT Certification Portal. https://certificate.fiesta-iot.eu) as many times as necessary, these tools were used for the tuning and polishing of their annotators and TPS implementations. The key aspect that was necessary to highlight to the TPs was precisely that they should use the certification process for their benefit instead of thinking that it is a hurdle to be overcome.

Specific technical details: Before the TPs are provided with credentials to register their testbeds at the FIESTA-IoT Platform, they have to obtain a certificate that the previous steps have been accomplished correctly. The Certification Portal basically offers a set of tools for validating the annotations and the TPS interfaces that the underlying testbed is offering. It is important to highlight that it is recommended, as a best practice for the TPs, to exploit the tools available at the Certification Portal during the development of their TPS interfaces and annotators and not restrict its use to the pure certification. In this sense, TPs have an unlimited number of attempts at the Certification Portal and it provides assessment reports on each attempt. Thus, the TPs are able to progressively fine tune the annotations and TPS implementation according to the reports obtained.

For the validation of the annotators, the Certification Portal provided a generic semantic validator that assessed both the syntactic (i.e., literals, URI and namespaces validation) and semantic (i.e., predicate and class validation and semantic errors) analysis of the RDF documents provided.

For the TPS validation, the Portal had an interoperability testing tool which included tests for all the possible TPS interfaces. TPs can provide the details of their TPS (endpoints, sensor identifiers, API Key, etc.) and check if they behave as the FIESTA-IoT Platform expects.

#### 5.1.2. Integrating the Testbed

Once the testbed is certified, it is now ready to be integrated to the FIESTA-IoT platform. As described before, the testbed integration process consists on a number of steps which requires actual interaction with FIESTA-IoT Platform interfaces. All the steps have been streamlined to make then user-friendly. However, there are still best practices and lessons to be learnt that can only be acquired through actually taking the aforementioned steps. In this sense, all the steps have been briefly introduced at the beginning of this section and for each of them there is a specific aspect that, from the experience that we have acquired, must be carefully addressed. Thus, during the registration of testbed and devices, which is made through a graphical user interface, the testbed provider must take care of the correct alignment of testbed and resources description as the linked data paradigm is used within the FIESTA-IoT Platform and consistent information is critical in order to exploit the potential of this paradigm. For the configuration of the TPS, even when functional interoperability of interfaces had been previously certified, finding the most suited schedule is not always straightforward. Finally, confirming that the integration has been properly carried out and it is properly maintained in time (testbed integration is a continuous process) requires the execution of a number of tests that qualify this integration.

Challenges and mitigation: In order to allow the testbed providers to try out their deployment and fix issues incrementally, the FIESTA-IoT platform provides a controlled test environment. The challenges described in this section were tackled within the test environment, and had no impact on the experimenters, who interact with the actual production environment where the testbeds are only deployed when stable. The entire integration process is first performed in this test environment before being made public.

##### Registering the Testbed and Its Resources

The first step to the testbed deployment is its registration, where some information about the testbed as a whole are provided, and in particular the endpoint of its TPS. Once the testbed is declared, its resources can be registered as it is described above. At this point, the resources of the testbed can be discovered by the experimenters, but no observation data has still been published.

Challenges and mitigation: Creating the annotated descriptions of the testbed devices can be a tough and complicated task mostly for providers that have little or no expertise with semantics and its peculiarities. Moreover, some of the testbeds had a large number of devices which could make this process time-consuming. The AaaS tool that was provided to the testbeds transforms this potentially complex step into a more user-friendly process in which testbed providers simply creates plain JSON documents from a simple template and through a single API call they can generate the descriptions from all the resources in their testbeds.

##### Configuring the TPS

The platform implements a tool to configure its behavior regarding the TPS. If the data is pushed by the TPS, the platform declares the resources it is interested in, and the TPS pushes data observed by these sensors as soon as it is available. Otherwise, the platform collects data from the platform at fixed intervals.

Challenges and mitigation: While configuring the TPS one challenge that occurs is that not all resources can be scheduled at a single time. This usually happens when a testbed has thousands of resources. To schedule all the resources it is advised that resources should be grouped in different chunks where each chunk has a different schedule to reduce the load of each individual push. Another issue is the impossibility to edit a schedule once it is registered. This makes it tedious for large testbed if some resources are not reliable, and require to be removed from the platform schedule individually.

#### 5.1.3. Running the TPS and Publishing Data to the Platform

At this point in the integration process, the testbed is compliant in every aspects with the FIESTA-IoT specification, and the data it generates can be consumed by the experimenters. The FIESTA-IoT platform acts as a data broker: there is no direct communication between the data producers and consumers, they only communicate with the platform through standardized interfaces.

Challenges and mitigation: Some challenges are faced when the TPS is running, especially in the virtual environment. One of the issues is the size of the bulk loads: the data that is transferred via TPS to the platform as RDF/XML. This data can be quite verbose leading to documents that are sometimes too large to be reliably transferred over the network. In order to keep the platform side as generic as possible, the mitigation strategies for this issue have to be implemented by the testbeds. In order to limit overload, the publications of data to the platform must be paced, and a 5-seconds break between the pushes ensures that the pushed data has been completely processed before publishing new data. Moreover, the datasets can be split into smaller subsets before being pushed to the platform. Once integrated to the knowledge graph on the platform’s side, this bursting at publication type has not impact on the experimentations. Another issue is the lack of synchronism between the data collection and publication: depending on the testbeds inner configuration, data can only be published sometime after having been collected. This interval can be of several hours, up to days.

#### 5.1.4. Technical Integration Concluding Remarks

**Figure 9:**
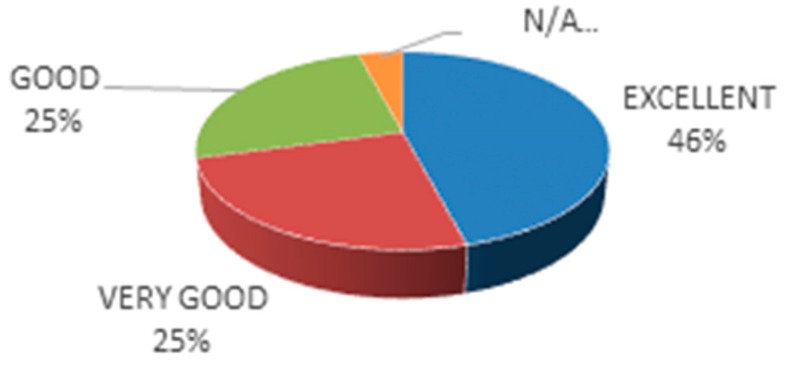
Evaluation of the testbed integration. Assessment of ease of setting up and deployment.

The evaluation of the testbed integration process was twofold. On the one hand, it was based on feedback provided by the Testbed Providers. The feedback focused on the assessment of the requirements, and the effort to address them, (like annotation process, certification of their testbeds, and interfaces implementation) in order to complete the testbed integration. As it is shown in Figure 9, most of them agreed that the ease of deployment was above satisfactory and they did not experience any significant degradation in terms of performance within their testbeds. This was expected as the integration process, as it has been presented above does not alter any of the internal procedures of the testbeds.

On the other hand, a non-functional evaluation was carried out focusing on the response times that the platform offered to the underlying testbeds upon they sent their observations for its storage into the FIESTA-IoT Platform.

**Figure 10:**
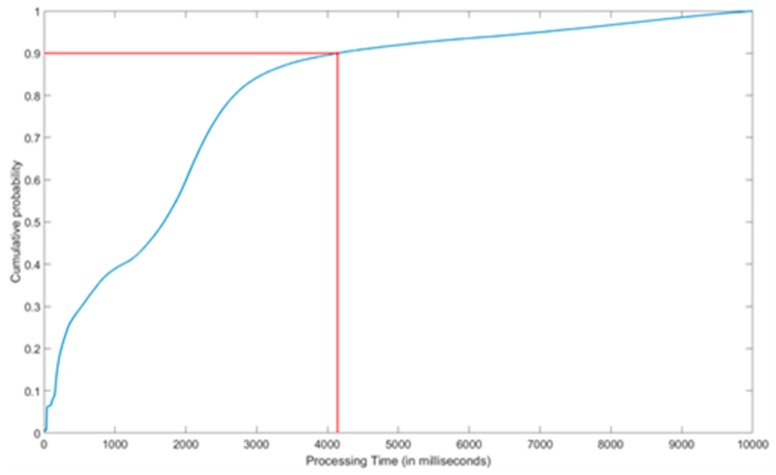
Cumulative Distribution Function of processing times for observations’ writings.

**Figure 11:**
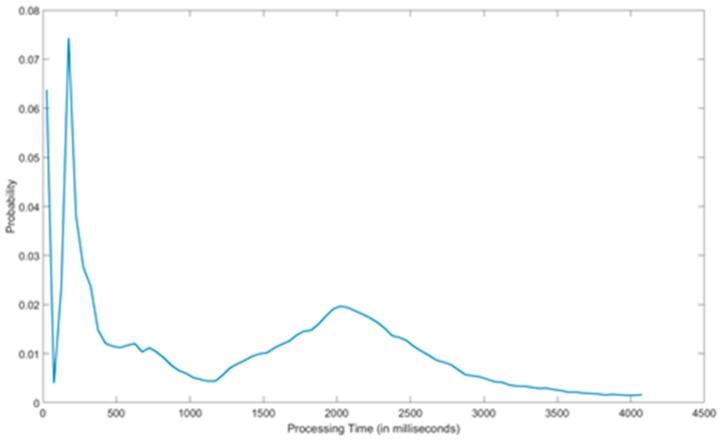
Probability Density Function of processing times for observations’ writings.

Figure 10 and Figure 11 shows the results from the performance analysis made. As it can be seen, 90% of the queries are handled in less than 4.1 seconds while the amount of queries taking more than 8 seconds is negligible. Moreover, it is interesting to highlight that more than half of the queries were responded in less than 2 seconds. In that sense, we can notice that the distribution is not completely exponential, and most probable processing times are around 10, 200 and 2000 milliseconds. In this respect it is important to remind that testbeds were asked not to push individual observations but to digest a number of them into each query. The amount of observations per query were dependent on the size of the testbed and the nature of the observations. In this sense, smaller testbeds typically digested all its observations (up to a couple of tens of them) in each query, while larger ones used a digest size of 100 observations per query. Thus, the response times show this heterogeneity as it can be clearly seen in Figure 11. However, even in the case of this larger batches of observations the response time of the system was rather good. Note that in its semantic representation every observation is composed of 7 objects.

**Figure 12:**
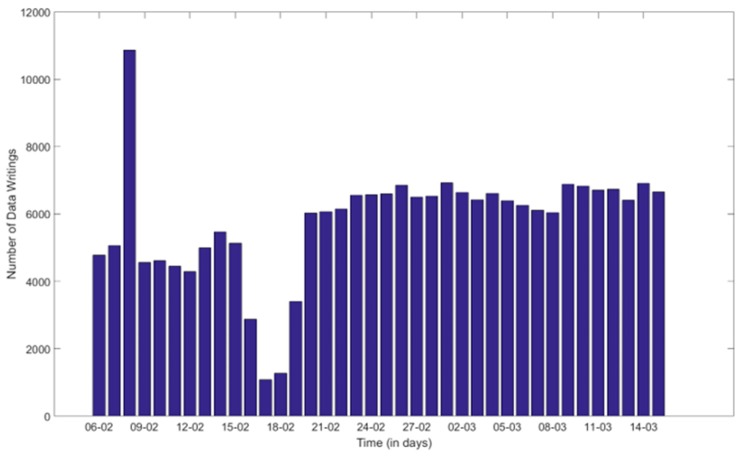
Observations’ writings per day during the analyzed period.

It is important to highlight that these results have been obtained under heavy load in terms of the demand that the system was experiencing. As it is presented in [16], the performance evaluation was carried out between 6 February 2018 and 15 March 2018. At that time, the amount of queries that the FIESTA-IoT platform received each day was, in average, around 13,000 queries per day, which is equivalent to 9 queries per minute. The FIESTA-IoT platform was not only storing the observations coming from the 11 testbeds but also serving the experimenters’ demands at the same time. From the total amount of queries, Figure 12 presents the number of data writings (i.e., the amount of queries for storing observations) coming from the underlying testbeds.

As it can be seen, there were a massive and continuous flow of observations coming from the 11 testbeds towards the FIESTA-IoT Platform.

### 5.2. Federation Exploitation Discussion

The FIESTA-IoT federation has been currently joined by eleven heterogeneous IoT facilities spanning a wide range of application domains. In this section, we discuss the motivation behind joining the testbed federation, both for the testbeds and the affiliated organizations.

The key offering of FIESTA-IoT platform is a common set of Testbed tools and APIs enabling collection of data from any of the federated testbeds. The unified approach of instrumenting experiments and collecting data through a single point of access and set of tools offers unique ease of experimentation over the wide variety of supported testbeds. Developers who want to use existing platforms need to negotiate access individually and adapt to the platform-specific API and information models. Having to perform these actions for each platform often limits the applicability of the developed applications as they have to be tailored to the different platforms. This fragmentation of the IoT and the missing interoperability result in high entry barriers for developers. Considering the complex case of experimenters interested in simultaneously accessing data stemming from multiple testbeds, we understand the advantage of employing the federation that inherently supports such scenarios, through the orchestration of a single experimental description.

The federation driven ease of use and ability to offer open data access generate direct advantages for connected testbeds, such as the expanded user base and platform visibility. In addition, the accumulated access of external users through the common platform portal has the potential to introduce users to new interesting testbeds and resources and further increase the individual testbed community. The federation also attracts totally new types of users that are not directly interested in the underlying facilities, but specifically in the applied semantic technologies, potentially searching for semantic interoperability with their data or application. The increased platform visibility offers also the potential for creating synergies between academic experimenters and industrial developers.

At the heart of the federation-enabling mechanisms lies the common semantic ontology, which serves as means for linking related data streams and ensuring interoperability of data stemming from totally heterogeneous resources. The federation includes testbeds like CABIN and ADREAM that had previously incorporated semantic technologies in their platform operation, which had to work towards aligning their existing taxonomy and vocabulary with the FIESTA-IoT ontology. On the other hand, facilities like MARINE and RealDC had their first experience in implementing common standardized semantics, by joining the federation. Such developments directly benefited the testbeds adopting semantic technologies to query their resources more effectively and layer sophisticated analytics on top of collected data. In addition, the adoption of M3-Lite taxonomy by the FIESTA-IoT ontology has offered semantic interoperability with other testbeds too. Summing up, we remark that all federated testbeds gained significant experience in working with semantically annotated data and applying semantic technologies in-field, which can be beneficial for future projects as well.

Moreover, there are also other important benefits that must not be neglected. Firstly, the great dissemination opportunities, stemming from the overall project’s promotion and marketing activities, which offer increased visibility to all federated testbeds and affiliated organizations. Secondly, the ability of all participating members to directly strengthen their position in the IoT and testbed experimentation communities, resulting in increased qualification for attracting additional funding through relevant research initiatives (EU research calls, etc.) or through potential organizations interested in using the testbed resources and offered measurements.

Last but not least, FIESTA-IoT Platform has proven successful in realizing the open data concept that FIESTA-IoT/EU is supporting in order to democratize the access to data. Not just in the sense of simply opening the data but increasing the meaningfulness of this data as the datasets are upgraded through the interoperability feature that the FIESTA-IoT Platform design and implementation intrinsically conveys.

## 6. Conclusions

Supporting real-world experimentation is undoubtedly part of the innovation cycle for any technological advance. In view of the attention that the IoT is attracting, several IoT open infrastructures have been deployed all around the world. Even if the usefulness of these experimental infrastructures in their own right is out of any doubt, the ecosystem that they have created is scattered and domain specific. It is deemed necessary to offer a homogeneous and interoperable framework that can actually fulfil the requirements from IoT research and innovation communities. These communities are in need of an IoT data marketplace that can serve them with cross-domain, interoperable, real-world data and environments.

In this paper, we have described the instantiation of such an environment stemming through the federation of eleven different IoT deployments. The resulting environment offers, to the best of our knowledge, the highest level of diversity and scale available through any single or federated IoT experimentation infrastructure, integrating totally heterogeneous application domains (e.g., smart cities, maritime, smart building, crowd-sensing, smart grid, etc.) and offering over 10,000 IoT devices. The paper briefly introduces, for the sake of completeness, the high-level architecture and key technologies employed for the realization of this unique federation of heterogeneous IoT infrastructures. In addition, the key paper contribution is presented through the analysis of the insightful summary of the actual data offerings and key characteristics of all the testbeds that are currently federated. For the community of IoT-based innovators and developers it is of utmost importance to be acquainted with the real offerings stemming from the unparalleled ecosystem for IoT experimentation that the interoperable federation of testbeds described in the paper is making available [82].

Moreover, the paper summarizes first hand experiences, lessons learnt and best practices that have been elicited during the integration of the testbeds. The description of the challenges faced during the integration process and their mitigations is followed by a discussion on the benefits for the TPs who joined the federation. The discussion included in the paper condenses the views of the different testbed providers and, apart from describing the techniques employed to fulfil the technical challenges associated with the federation of heterogeneous IoT platforms, it should be extremely valuable for other infrastructure owners that might consider joining the current federation of testbeds.

In order to highlight the value of the contributions of this paper, it is important to mention that the platform design represents the necessary condition for supporting IoT experimentation, as it enables the dynamic discovery of resources and their data. However, it is only through the actual federation of testbeds described in the paper that the sufficient condition is met. Thus, we remark the significant value of our work for the IoT research community, since it is essentially the federation of testbeds the one that tangibly (i.e., not as an academic exercise but in the real-world) enables the dynamic on-demand formulation of cloud-based IoT services over a virtualized Data Marketplace.

The contributions of the paper are the description of the resulting federation of testbeds and the lessons learnt during the process of federating one testbed. Details of the actual platform and insights about the technical details about the platform that supports the federation are out of the scope of the paper. However, the semantically-enabled multi-domain data marketplace can be accessed on-line. In this sense, the testbed federation itself is, implicitly, the proof for the realization of this concept. Through the tools available from the FIESTA-IoT Platform (FIESTA-IoT Platform. https://platform.fiesta-iot.eu. [Online 3rd September 2018]), the experimenters can discover and access the whole variety of datasets and develop their research or innovative applications. The experimenters are able to discover the offerings from the data marketplace independently of the origin of the data. They only have to follow the FIESTA-IoT ontology while browsing and consuming the data that the underlying testbeds have previously pushed.

Future lines of development will include the development of cross-domain data analytics. Having the ability to apply data mining or machine learning techniques on top of the federation presented in this paper will have a twofold benefit. On the one hand, analytics developers benefit from the homogeneous access to heterogeneous datasets, thus, maximizing the re-utilization of the developed algorithms and minimizing the burden of having access to information that nowadays are part of different vertical. On the other hand, re-use of the techniques and services (like dashboards, KPIs derivation) developed for any of the testbeds federated boosts the attractiveness of the federation itself. In particular, data quality and infrastructure performance insights should be of great interest to IoT infrastructure providers who, in the majority of the cases, are experts in managing the physical infrastructure but does not have the know-how to effectively manage and curate the data that the infrastructure is producing and, more important, their customers consuming.

## Figures and Tables

**Table 1:** FIESTA-IoT testbeds summary.

Testbed	Short Description	Deployed Devices
*SmartSantander* [72]	Large-scale Smart City deployment.	Thousands of fixed and mobile sensors (environment, parking, transportation, etc.).
*SmartICS* [73]	Smart Environment based on an indoor deployment of sensor nodes.	Hundreds of indoor environment sensors.
*SoundCity* [74]	Crowdsensing testbed using mobile phones	Variable number of phone-based sensors measuring noise pollution and proximity.
*CABIN* *	Indoor and outdoor environment. Smart building deployment with outdoor sensors.	Hundreds of indoor environmental sensors with tens of outdoor parking sensors.
*NITOS* [75]	Heterogeneous Lora and Wireless Sensor Network.	20 LoRa and 60 Zigbee indoor environmental and presence sensors.
*MARINE* **	Seawater and Air quality monitoring testbed.	4 floating seawater quality monitoring buoys and 5 fixed air quality monitoring stations (17 different sensor types).
*RealDC*	Live data center testbed for monitoring DC operations.	100 sensors for power consumption and weather station producing over 2000 observations.
*Tera4Agri*	Outdoor testbed for Smart Agriculture.	More than 10 sensors for environmental, soil and tree monitoring.
*FINE*	Smart City, smart building and home automation testbed.	40 outdoor environmental monitoring and 6 indoor automation sensors and actuators.
*Grasse Smart Territory*	Smart City testbed open to local developer community who bring their own sensors	5 sensor boxes with each containing multi environmental sensors.
*ADREAM*	Large-scale smart building testbed	6500 sensors for lighting, electricity, HVAC, solar panels, etc.

* CABIN testbed. http://developers.iotocean.org [Online 13 August 2018]; ** GRIDNET. http://gridnet.gr/MARINE/ [Online 13 August 2018].

**Table 2:** Categorization of testbeds in different application domain.

Application Domain	Testbeds
Smart City	SmartSantander, SoundCity, CABIN, FINE, Grasse Territory
Smart Agriculture	Tera4Agri
Smart Buildings	SmartICS, CABIN, NITOS, FINE, ADREAM
Smart Energy	SmartICS, RealDC, ADREAM
Smart Sea	MARINE

## Appendix A. FIESTA-IoT Testbeds Detailed Offering

**Table A1:** FIESTA-IoT testbeds detailed offering.

Testbed	Phenomenon	FIESTA-IoT Quantiy Kind	Avg. Number of Active Sensors	Avg. Number of Observations Per Hour (Aprox.)
*SmartSantander*	Parking Availability	m3-lite#PresenceStateParking	150	n/a
	Air Temperature	m3-lite#AirTemperature	144	12
	Air Dust	m3-lite#ChemicalAgentAtmosphericConcentrationAirParticles	74	50
	CO	m3-lite#ChemicalAgentAtmosphericConcentrationCO	74	50
	NO_2_	m3-lite#ChemicalAgentAtmosphericConcentrationNO2	74	50
	O_3_	m3-lite#ChemicalAgentAtmosphericConcentrationO3	74	50
	Relative Humidity	m3-lite#RelativeHumidity	17	12
	Noise	m3-lite#SoundPressureLevelAmbient	11	12
	2.4 GHz Electromagnetic Field	m3-lite#ElectricField2400MHz	10	12
	2.1 GHz Electromagnetic Field	m3-lite#ElectricField2100MHz	10	12
	1.8 GHz Electromagnetic Field	m3-lite#ElectricField1800MHz	10	12
	900 MHz Electromagnetic Field	m3-lite#ElectricField900MHz	10	12
	Soil Humidity	m3-lite#SoilMoistureTension	8	12
	Soil Temperature	m3-lite#SoilTemperature	8	12
	People Count	m3-lite#CountPeople	7	6
	Waste Bin Fill Level	m3-lite#FillLevelWasteContainer	4	6
	Atmospheric Pressure	m3-lite#AtmosphericPressure	1	12
	Solar Radiation	m3-lite#SolarRadiation	1	12
	Wind Speed	m3-lite# WindSpeed	1	12
	Wind Direction	m3-lite# WindDirection	1	12
*SmartICS*	Building Temperature	m3-lite#Temperature and m3-lite#RoomTemperature	104	6
	Relative Humidity	m3-lite#Humidity	104	6
	Noise	m3-lite#Sound	103	6
	Illuminance	m3-lite#Illuminance	103	6
	People Presence	m3-lite#Distance	98	6
	Active Power Consumption	m3-lite#Power	29	6
*SoundCity*	Noise	m3-lite#Sound	4	n/a
	Direction Heading	m3-lite#DirectionHeading	4	n/a
	Presence	m3-lite#Proximity	4	n/a
	Average Speed	m3-lite#SpeedAverage	4	n/a
*CABIN*	People Presence	m3-lite#PresenceStatePeople	49	n/a
	Building Temperature	m3-lite#BuildingTemperature	41	6
	Relative Humidity	m3-lite#RelativeHumidity	41	6
	Illuminance	m3-lite#Illuminance	39	6
	Parking Availability	m3-lite#PresenceStateParking	19	n/a
	CO_2_	m3-lite#CO2	10	6
	Active Power Consumption	m3-lite#Power	9	6
*NITOS*	People Presence	m3-lite#PresenceStatePeople	4	3
	Building Temperature	m3-lite#AirTemperature	3	3
	Relative Humidity	m3-lite#Humidity	3	3
	Illuminance	m3-lite#WeatherLuminosity	3	3
	Noise	m3-lite#SoundPressureLevel	2	3
	Door Status	m3-lite#DoorStatus	2	3
	Radiation	m3-lite#IonisingRadiation	1	3
*MARINE*	Sea Water PH	m3-lite#PH	3	4
	Sea Water Temperature	m3-lite#WaterTemperature	3	4
	Sea Water Conductivity	m3-lite#Conductivity	2	4
	Sea Water Oxidation Reduction	m3-lite#Voltage	2	4
	Atmospheric Pressure	m3-lite#AtmosphericPressure	2	6
	Air Temperature	m3-lite#AirTemperature	2	6
	Relative Humidity	m3-lite#Humidity	2	6
	Water NO_3_ Ion	m3-lite#ChemicalAgentWaterConcentrationNO3Ion	1	4
	IEEE 802.15.4 Signal Level	m3-lite#Power	1	6
	IEEE 802.11 Signal Level	m3-lite#Power	1	6
	LoRa Device RSSI	m3-lite#Power	1	6
*RealDC*	Electric Voltage	m3-lite#Voltage	486	4
	Electric Current	m3-lite#ElectricCurrent	243	4
	Active Power Consumption	m3-lite#ActivePower	81	4
	Reactive Power	m3-lite#ReactivePower	81	4
	Electric Frequency	m3-lite#Frequency	81	4
	Air Temperature	m3-lite#AirTemperature	33	3
	Cooling Water Temperature	m3-lite#WaterTemperature	32	3
	Atmospheric Pressure	m3-lite#AtmosphericPressure	1	4
	Dew Point	m3-lite#DewPointTemperature	1	4
	Relative Humidity	m3-lite#RelativeHumidity	1	4
	Rainfall	m3-lite#Rainfall	1	4
	Wind Chill	m3-lite#WindChill	1	4
	Wind Speed	m3-lite# WindSpeed	1	4
	Wind Direction	m3-lite# WindDirection	1	4
*Tera4Agri*	Soil Humidity	m3-lite#SoilHumidity	9	2
	Soil Temperature	m3-lite#SoilTemperature	1	2
	Air Temperature	m3-lite#AirTemperature	1	2
	Dew Point	m3-lite#DewPoint	1	2
	Leaf Weatness	m3-lite#LeafWetness	1	2
	Rainfall	m3-lite#Precipitation	1	2
	Relative Humidity	m3-lite#RelativeHumidity	1	2
	Solar Radiation	m3-lite#SolarRadiation	1	2
	Wind Speed	m3-lite#WindSpeed	1	2
	Wind Direction	m3-lite#WindDirection	1	2
*FINE*	Board Temperature	m3-lite#BoardTemperature	20	6
	Board Voltage	m3-lite#Voltage	20	6
	Device Uptime	m3-lite#DeviceUptime	20	6
	IEEE 802.15.4 Signal Level	m3-lite#Power	20	6
	Air Temperature	m3-lite#AirTemperature	17	6
	Relative Humidity	m3-lite#RelativeHumidity	17	6
	Air Dust	m3-lite#ChemicalAgentAtmosphericConcentrationAirParticles	12	6
	Illuminance	m3-lite#Illuminance	11	6
	Noise	m3-lite#SoundPressureLevelAmbient	10	6
	Electric Current	m3-lite#ElectricCurrent	7	6
	Electric Voltage	m3-lite#Voltage	2	6
	NO	m3-lite#ChemicalAgentAtmosphericConcentrationNO	2	6
	NO_2_	m3-lite#ChemicalAgentAtmosphericConcentrationNO2	2	6
	CO_2_	m3-lite#CO2	2	6
	O_3_	m3-lite#ChemicalAgentAtmosphericConcentrationO3	2	6
	SO_2_	m3-lite#ChemicalAgentAtmosphericConcentrationSO2	2	6
	VOC	m3-lite#ChemicalAgentAtmosphericConcentrationVOC	2	6
*Grasse Smart Territory*	Lora Device SNR	m3-lite#SNR	7	10
	Lora Device RSSI	m3-lite#RSSI	7	10
*ADREAM*	Electric Voltage	m3-lite#Voltage	145	8
	Building Temperature	m3-lite#Temperature	54	3
	Air Temperature	m3-lite#AirTemperature	36	2
	Electric Power	m3-lite#Energy	10	10
